# Brain region-specific altered expression and association of mitochondria-related genes in autism

**DOI:** 10.1186/2040-2392-3-12

**Published:** 2012-11-01

**Authors:** Ayyappan Anitha, Kazuhiko Nakamura, Ismail Thanseem, Kazuo Yamada, Yoshimi Iwayama, Tomoko Toyota, Hideo Matsuzaki, Taishi Miyachi, Satoru Yamada, Masatsugu Tsujii, Kenji J Tsuchiya, Kaori Matsumoto, Yasuhide Iwata, Katsuaki Suzuki, Hironobu Ichikawa, Toshiro Sugiyama, Takeo Yoshikawa, Norio Mori

**Affiliations:** 1 Research Center for Child Mental Development, Hamamatsu University School of Medicine, 1-20-1 Handayama, Hamamatsu, 431 3192, Japan; 2 Department of Psychiatry and Neurology, Hamamatsu University School of Medicine, 1-20-1 Handayama, Hamamatsu, 431 3192, Japan; 3 Laboratory for Molecular Psychiatry, RIKEN Brain Science Institute, 2-1 Hirosawa, Wako, 351 0198, Japan; 4 Tokyo Metropolitan Children’s Medical Center, 2-8-29 Musashidai, Fuchu, 183 8561, Japan; 5 Faculty of Sociology, Chukyo University, 101 Tokodachi, Toyota, 470 0393, Japan; 6 Department of Child and Adolescent Psychiatry, Hamamatsu University School of Medicine, 1-20-1 Handayama, Hamamatsu, 431 3192, Japan

**Keywords:** Autism, Mitochondria, Postmortem brain, NEFL, Uncoupling protein, Metaxin

## Abstract

**Background:**

Mitochondrial dysfunction (MtD) has been observed in approximately five percent of children with autism spectrum disorders (ASD). MtD could impair highly energy-dependent processes such as neurodevelopment, thereby contributing to autism. Most of the previous studies of MtD in autism have been restricted to the biomarkers of energy metabolism, while most of the genetic studies have been based on mutations in the mitochondrial DNA (mtDNA). Despite the mtDNA, most of the proteins essential for mitochondrial replication and function are encoded by the genomic DNA; so far, there have been very few studies of those genes. Therefore, we carried out a detailed study involving gene expression and genetic association studies of genes related to diverse mitochondrial functions.

**Methods:**

For gene expression analysis, postmortem brain tissues (anterior cingulate gyrus (ACG), motor cortex (MC) and thalamus (THL)) from autism patients (n=8) and controls (n=10) were obtained from the Autism Tissue Program (Princeton, NJ, USA). Quantitative real-time PCR arrays were used to quantify the expression of 84 genes related to diverse functions of mitochondria, including biogenesis, transport, translocation and apoptosis. We used the delta delta Ct (∆∆Ct) method for quantification of gene expression. DNA samples from 841 Caucasian and 188 Japanese families were used in the association study of genes selected from the gene expression analysis. FBAT was used to examine genetic association with autism.

**Results:**

Several genes showed brain region-specific expression alterations in autism patients compared to controls. Metaxin 2 (*MTX2*), neurofilament, light polypeptide (*NEFL*) and solute carrier family 25, member 27 (*SLC25A27*) showed consistently reduced expression in the ACG, MC and THL of autism patients. *NEFL* (*P* = 0.038; Z-score 2.066) and *SLC25A27* (*P* = 0.046; Z-score 1.990) showed genetic association with autism in Caucasian and Japanese samples, respectively. The expression of *DNAJC19*, *DNM1L*, *LRPPRC*, *SLC25A12*, *SLC25A14*, *SLC25A24* and *TOMM20* were reduced in at least two of the brain regions of autism patients.

**Conclusions:**

Our study, though preliminary, brings to light some new genes associated with MtD in autism. If MtD is detected in early stages, treatment strategies aimed at reducing its impact may be adopted.

## Background

Autism is a complex neurodevelopmental disorder characterized by deficiencies in social interaction and communication, and repetitive and stereotyped behaviors. Autistic disorder, Asperger syndrome, and pervasive developmental disorder-not otherwise specified (PDD-NOS) comprise a heterogeneous group of neurodevelopmental disorders known as autism spectrum disorders (ASD). The abnormalities are usually identified in the early years of childhood and often coexist with impairments in cognitive functioning, learning, attention and sensory processing. According to a recent report, the prevalence of this disorder has risen to 1 in 110, with a male to female ratio of 4.5:1 [1].

A growing body of evidence from biochemical and neuroimaging studies has suggested that a disturbed brain bioenergetic metabolism underlies the pathophysiology of autism in some cases. Magnetic resonance spectroscopy studies have shown, in the brain of autism patients, abnormal levels of metabolites relating to brain bioenergetics, such as decreased levels of phosphocreatine and *N*-acetyl-aspartate, and elevated lactate [2,3].

Mitochondria serve as the energy powerhouses of eukaryotic cells, since they generate most of the adenosine triphosphate (ATP), the source of chemical energy in cells. The findings of abnormal brain bioenergetics, therefore, support an involvement of mitochondrial dysfunction (MtD) in the pathogenesis of ASD [4]. Diminished levels of ATP have been observed in autism brain [2]. Rats induced for MtD have been found to exhibit certain brain, behavioral and metabolic changes consistent with ASD, including microglial activation, reduced levels of glutathione, repetitive behaviors, social interaction deficits, hyperactivity and oxidative stress (OS) [5-8].

In a systematic review and meta-analysis, MtD was observed in approximately five percent of children with ASD; developmental regression, seizures, motor delay and gastrointestinal abnormalities were found to be significantly more prevalent in children with ASD/MtD as compared with the general ASD population [9]. Defective lymphocytic mitochondria [10] and ultrastructural abnormalities of mitochondria [11,12] have been reported in autism. Nutritional supplements (for example, carnitine, vitamin B) and/or antioxidants (for example, co-enzyme Q10) have been found to be beneficial in the treatment of some children with ASD/MtD [13-15].

Recent studies have reported brain region-specific deficits of mitochondrial electron transport chain complexes in autism [16,17]. Upregulated expression of the mitochondrial aspartate/glutamate carrier (SLC25A12) [18,19], and evidence of hypoxia, as measured by a reduction in the anti-apoptotic protein Bcl-2 and an increase in the pro-apoptotic protein p53 [20,21], has also been reported in autism brain.

Several of the previous studies of MtD in autism were restricted to the biomarkers of energy metabolism, while most of the genetic studies were based on mutations in the mitochondrial DNA (mtDNA). Despite the mtDNA, most of the proteins essential for mitochondrial replication and function are encoded by the genomic DNA; so far, there have been very few studies of those genes. We aimed at elucidating the role of MtD in the pathogenesis of autism. Using the postmortem brains of autism patients and healthy controls, we compared the expression of 84 genes involved in diverse functions of the mitochondria such as, biogenesis, transport, translocation and apoptosis. Furthermore, we analyzed the genetic association of three of these genes with autism, in two independent studies involving family-based samples of different origins.

## Methods

### Gene expression studies of human postmortem brains

#### Postmortem brain tissues

Postmortem brain samples of autism patients and healthy controls were provided by the Autism Tissue Program (http://www.autismtissueprogram.org), NICHD Brain and Tissue Bank for Developmental Disorders (NICHD BTB; Baltimore, MD, USA; http://medschool.umaryland.edu/btbank/) and Harvard Brain Tissue Resource Center (HBTRC; Belmont, MA; http://www.brainbank.mclean.org/). Frozen tissue samples from anterior cingulate gyrus (ACG), motor cortex (MC) and thalamus (THL) were used in the study. Demographic characteristics of the samples (ACG: eight autism, ten controls; MC: seven autism, eight controls; THL: eight autism, nine controls) are described in Table 1.

**Table 1:** Postmortem brain tissue information

**Sample ID**^**a**^	**Diagnosis**	**Age (years)**	**Gender**	**PMI (hours)**	**Race**	**Cause of death**	**Brain regions**^**b**^
1065	Control	15	M	12	Caucasian	Multiple injuries	ACG, THL
1297	Control	15	M	16	African American	Multiple injuries	ACG, MC, THL
1407	Control	9	F	20	African American	Asthma	ACG, MC, THL
1541	Control	20	F	19	Caucasian	Head injuries	ACG, MC, THL
1649	Control	20	M	22	Hispanic	Multiple injuries	ACG, MC, THL
1708	Control	8	F	20	African American	Asphyxia, multiple injuries	ACG, MC, THL
1790	Control	13	M	18	Caucasian	Multiple injuries	ACG
1793	Control	11	M	19	African American	Drowning	ACG, MC, THL
1860	Control	8	M	5	Caucasian	Cardiac Arrhythmia	ACG
4543	Control	28	M	13	Caucasian	Multiple injuries	MC, THL
4638	Control	15	F	5	Caucasian	Chest injuries	ACG
4722	Control	14	M	16	Caucasian	Multiple injuries	MC, THL
797	Autism	9	M	13	Caucasian	Drowning	ACG, THL
1638	Autism	20	F	50	Caucasian	Seizure	ACG, MC, THL
4231	Autism	8	M	12	African American	Drowning	ACG, MC, THL
4721	Autism	8	M	16	African American	Drowning	ACG, MC, THL
4899	Autism	14	M	9	Caucasian	Drowning	ACG, MC, THL
5000	Autism	27	M	8.3	NA	NA	ACG, MC, THL
6294	Autism	16	M	NA	NA	NA	ACG, MC, THL
6640	Autism	29	F	17.83	NA	NA	ACG, MC, THL

^a^Autism Tissue Program (ATP) identifier; ^b^Brain regions for which, each sample was available; ACG, anterior cingulate gyrus; F, female; M, male; MC, motor cortex; NA, not available; PMI, postmortem interval; THL, thalamus.

The difference in age and postmortem interval (PMI) between the autism and control groups was examined by *t*-test. Fisher’s Exact test was used to examine the difference in sex distribution between the two groups (see Additional file 1).

#### RNA extraction

The brain tissues (approximately 75 mg obtained by macrodissection) were homogenized by ultrasonication, and total RNA was extracted using TRIzol™ Reagent (Invitrogen, Carlsbad, CA, USA) in accordance with the manufacturer’s protocol. The RNA samples were further purified using an RNeasy™ Micro Kit (QIAGEN GmbH, Hilden, Germany) following the manufacturer’s instructions; this protocol includes a DNase treatment step. The quantity (absorbance at 260 nm) and quality (ratio of absorbance at 260 nm and 280 nm) of RNA were estimated with a NanoDrop ND-1000 Spectrophotometer (Scrum, Tokyo, Japan). As per the requirements for the subsequent array experiment, the following criteria were met for all of the RNA samples: 1) A260:A230 ratio, >1.7; 2) A260:A280 ratio, between 1.8 and 2.0 (the A260:A280 ratio of all our RNA samples were in the range of 2.0 to 2.1), and 3) concentration of total RNA, >40 ng/μl.

#### First strand cDNA synthesis

First-strand cDNA was synthesized from 500 ng of total RNA using the RT^2^ First Strand Kit (SABiosciences, Frederick, MD, USA) following the manufacturer’s protocol. The kit contains a genomic DNA elimination step and a built-in external RNA control that helps monitor reverse transcription efficiency and tests for contaminating inhibitors during quantitative PCR (qPCR).

#### qPCR

We used the RT^2^ Profiler^TM^ PCR Array Human Mitochondria (SABiosciences) for quantifying the expression of 84 genes related to the biogenesis and functions of mitochondria. The array also has five reference genes (beta-2-microglobulin (*B2M*), hypoxanthine phosphoribosyltransferase 1 (*HPRT1*), ribosomal protein L13a (*RPL13A*), glyceraldehyde-3-phosphate dehydrogenase (*GAPDH*), and actin beta (*ACTB*)), three reverse transcription controls (RTCs), three positive PCR controls (PPCs), and one genomic DNA control (GDC), making up to a total of 96 assays. The details of the genes are provided in Additional files 2 and 3. The 384-well format of the array includes four replicates of each of the 96 assays. It makes use of the SYBR^TM^ Green method of qPCR analysis. The qPCR reactions were carried out according to the manufacturer’s protocol, in ABI PRISM 7900HT SDS (Applied Biosystems (ABI), Foster City, CA, USA).

#### Data analysis

The threshold cycle (Ct) values obtained from qPCR were analyzed by the ∆∆Ct method using RT^2^ Profiler PCR Array Data Analysis (Microsoft Excel-based program of SABiosciences). It calculates:

1) ∆Ct of each gene = Ct of gene of interest - average Ct of chosen reference genes

2) ∆∆Ct for each gene across two groups; ∆∆Ct = ∆Ct (autism group) - ∆Ct (control group)

3) fold-change for each gene from control group to autism group as 2 ^ (−∆∆Ct)

Based on the Kolmogorov-Smirnov test, the expression of all genes was found to follow a normal distribution. Therefore, a *t-*test, which was also the default option in our data analysis program, was used to examine any significant difference in gene expression between the control and autism groups.

The statistical program also performs the following functions: 1) interprets all Ct values ≥35 as a negative call; 2) examines genomic DNA contamination in the samples based on the Ct of GDC (Ct <35 will indicate genomic DNA contamination); 3) examines the presence of impurities in RNA samples based on the Ct value of PPC (Ct should be 20 ± 2 on each array and should not vary by more than two cycles between the arrays being compared); and 4) interprets any inhibition of reverse transcription based on the Ct values of RTC and PPC (values <5 for Ct RTC - Ct PPC is indicative of no apparent inhibition).

### Genetic association study

An association study, rather than deep sequencing, is considered as a cost-effective approach for studying complex traits like autism.

#### AGRE subjects

841 pedigree samples (3211 individuals in total) were obtained from Autism Genetic Resource Exchange (AGRE; http://www.agre.org; Los Angeles, CA, USA) [22]. This includes 1467 patients (1178 males; 289 females) with autism. Pedigree information for each individual, along with the diagnoses based on Autism Diagnostic Interview-Revised (ADI-R) [23], are available in the AGRE website. Families with a non-idiopathic autism flag (for example, fragile-X, abnormal brain imaging results, dysmorphic features, birth trauma) recorded for any of its members were excluded from the study.

#### Japanese subjects

The aforementioned AGRE samples were predominantly of Caucasian origin. Therefore, Japanese samples were used in an effort to replicate the AGRE genetic association results in samples of a different ethnicity. The Japanese trio samples included 188 children with ASD [gender: 155 males, 33 females; age: 10.49 ± 4.75 years (mean ± SD); IQ: 82.06 ± 26.6] and both parents for each child. All of the subjects were Japanese, born and living in the areas of central Japan including Chukyo, Tokai and Kanto. The purpose of the study was fully explained to the participants, and written informed consent was obtained. This study was approved by the Ethics Committee of Hamamatsu University School of Medicine. The diagnosis of autism was based on ADI-R [23] and DSM-IV-TR [24]. We are licensed to privately use a Japanese version of ADI-R by Professor C. Lord. ADI-R scores were available for 100 patients. All of the autistic individuals underwent screening to exclude comorbid psychiatric illnesses (for example, schizophrenia, affective disorders, mental retardation, and personality or behavioral disorders) by means of the Structured Clinical Interview for DSM-IV (SCID) [25]. Individuals with a history of neurological disorders (for example, epilepsy, head injury) or genetic disorders (for example, fragile X syndrome, tuberous sclerosis) were excluded.

#### Single nucleotide polymorphism (SNP) selection

The genomic structures of metaxin 2 (*MTX2*; 2q31.1), neurofilament, light polypeptide 68 kDa (*NEFL*; 8p21.2), and solute carrier family 25, member 27 (*SLC25A27*; 6p12.3) were based on the University of California, Santacruz (UCSC; http://genome.ucsc.edu/) February 2009 draft assembly of the human genome. *MTX2* (68.626 kb) consists of 11 exons, *NEFL* (5.664 kb) of 4 exons, and *SLC25A27* (25.238 kb) of 9 exons. SNPs (minor allele frequency >0.1) for the association study were selected from the International HapMap Project (http://www.hapmap.org) database on Caucasian and Japanese populations. Additional file 4 gives the list of SNPs chosen for the three genes using the pairwise tagging option of Haploview v4.1 (http://www.broad.mit.edu/mpg/haploview).

#### Genotyping

Assay-on-Demand/Assay-by-design SNP genotyping products (ABI) were used to score genotypes, based on the TaqMan™ assay method [26]. Genotypes were determined in ABI PRISM 7900HT SDS (ABI), and analyzed using SDS v2.0 software (ABI).

#### Statistical analysis

FBAT v2.0.3 (http://biosun1.harvard.edu/~fbat/fbat.htm) was used to examine the associations of SNPs with autism. FBAT-MM option was used for multimarker test. FBAT provides valid tests of association in the presence of linkage even when using multiple affected siblings from families of variable structure. In addition to performing tests of association for individual markers, FBAT allows for tests of association with haplotypes that may be phase ambiguous. Linkage disequilibrium (LD) plots based on D′ values were constructed using Haploview. Haplotype association was also examined using this software. Power analysis was done using the Genetic Power Calculator (http://pngu.mgh.harvard.edu/~purcell/gpc/dtdt.html).

## Results

### Gene expression analysis using human postmortem brain samples

There was no significant difference in age, postmortem interval (PMI) or sex distribution between the autism and control groups in any of the brain regions analyzed (see Additional file 1).

In the qPCR experiment, genomic DNA contamination was not observed for any of the RNA samples; Ct GDC was >35 for all the samples, indicating that genomic DNA contamination, if present, was too low to affect the gene expression results. Ct PPC was 20 ± 2 for all the arrays compared, showing the apparent absence of impurities in the RNA samples. Further, there was no indication of any inhibition of reverse transcription for any of the samples, since Ct RTC - Ct PPC was <5 for all the samples.

For normalization of gene expression, the following reference genes were selected for the various brain regions: 1) ACG: *RPL13A*, *GAPDH* and *ACTB;* 2) MC: *B2M*, *RPL13A*, *GAPDH* and *ACTB;* 3) THL: *B2M*, *HPRT1* and *GAPDH*. The chosen reference genes for each brain region did not show any significant difference in expression between the control and autism groups.

We observed brain region-specific alterations in the expression of several genes in the autism group compared to the control group (Table 2). A total of 22 genes in ACG, 15 genes in MC and 12 genes in THL showed aberrant expression in autism patients compared to controls. These genes belong to the following functional groups: 1) membrane polarization and potential, 2) mitochondrial transport, 3) small molecule transport, 4) targeting proteins to mitochondria, 5) mitochondrion protein import, 6) outer membrane translocation, 7) inner membrane translocation, 8) mitochondrial fission and fusion, 9) mitochondrial localization, and 10) apoptosis (Table 2). A majority of the genes showed a reduced expression in autism as compared to the controls. Eleven genes each belonging to the SLC25A mitochondrial transporter family and TIMM/TOMM family of translocases, showed altered expression in autism.

**Table 2:** Genes with altered expressions in autism postmortem brains

**Gene**	**Anterior Cingulate Gyrus**		**Motor Cortex**		**Thalamus**	
	**Fold change**	*P*value^*^	**Fold change**	*P*value^*^	**Fold change**	*P*value^*^
*AIP*^b,d,e^			−1.473	0.048		
*BCL2*^a,b,j^	1.356	0.045				
*DNAJC19*^b,d,e^			−1.868	0.037	−1.520	0.030
*DNM1L*^i,j^	−1.658	0.020	−1.603	0.045		
*HSP90AA1*^b^					1.662	0.044
*LRPPRC*^i^	−1.486	0.025	−1.530	0.018		
*MFN2*^b,d,h,i^	−1.446	0.021				
*MIPEP*^b,d,e^			−2.003	0.012		
*MTX2*^b^	−1.795	0.044	−2.055	0.017	−2.511	0.002
*NEFL*^i^	−4.208	0.014	−2.935	0.025	−6.006	0.012
*RHOT2*^i^	−1.338	0.019				
*SLC25A12*^c^	−2.112	0.013	−1.913	0.008		
*SLC25A14*^c^	−1.876	0.034	−1.924	0.010		
*SLC25A15*^c^	−1.402	0.036				
*SLC25A22*^c^	−2.061	0.007				
*SLC25A24*^c^			−1.690	0.008	−1.625	0.019
*SLC25A25*^c^					1.830	0.044
*SLC25A27*^c^	−2.132	0.025	−2.167	0.042	−2.644	0.026
*SLC25A3*^c^			−1.668	0.020		
*SLC25A37*^c^					1.973	0.046
*SLC25A4*^c^	−1.614	0.034				
*SLC25A5*^c^			−1.548	0.027		
*SOD2*^j^					3.919	0.036
*TIMM17A*^g^	−1.809	0.011				
*TIMM17B*^g^	−1.601	0.006				
*TIMM23*^g^			−1.734	0.022		
*TIMM44*^g^					1.769	0.034
*TIMM50*^g^	−1.513	0.045				
*TIMM8A*^g^	−1.612	0.021				
*TIMM9*^g^					−1.940	0.010
*TOMM20*^f^	−1.812	0.025	−1.745	0.017		
*TOMM22*^f^	−1.450	0.024				
*TOMM34*^f^					−1.995	0.002
*TOMM70A*^f^	−1.525	0.041				
*TP53*^a,b,j^	1.769	0.021				
*TSPO*^b,d^	1.720	0.037				

^a^Membrane polarization and potential; ^b^Mitochondrial transport; ^c^Small molecule transport; ^d^Targeting proteins to mitochondria; ^e^Mitochondrion protein import; ^f^Outer membrane translocation; ^g^Inner membrane translocation; ^h^Mitochondrial fission and fusion; ^i^Mitochondrial localization; ^j^Apoptotic genes; ^*^P values were calculated by *t*-test.

**Figure 1:**
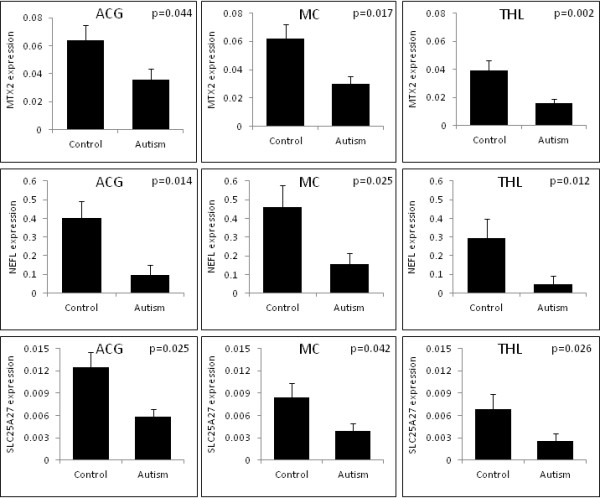
**Reduced expression of *MTX2*, *NEFL *and *SLC25A27 *in autism brain.** Comparison of the expression of *MTX2*, *NEFL* and *SLC25A27* in the various brain regions of autism patients and healthy controls. *MTX2*, *NEFL* and *SLC25A27*showed significantly reduced expression in all the three brain regions of autism patients. The *P* values (*t*-test) are given at the top right corner of each graph. The y-axis of each graph represents the relative expression of the respective gene normalized to the reference genes. The gene expression is normalized against the average Ct of the chosen reference genes for each brain region. The following reference genes were selected for the various brain regions: a) anterior cingulate gyrus (ACG): *RPL13A*, *GAPDH* and *ACTB* b) motor cortex (MC): *B2M*, *RPL13A*, *GAPDH* and *ACTB* c) thalamus (THL): *B2M*, *HPRT1* and *GAPDH*.

The genes *MTX2*, *NEFL* and *SLC25A27*showed consistently reduced expression in all the three brain regions (ACG, MC and THL) of autism patients (Figure 1). The most pronounced reduction, in autism brains, was observed for *NEFL* [−4.208 fold (*P* = 0.014) in ACG; -2.935 fold (*P* = 0.025) in MC; -6.006 fold (*P* = 0.012) in THL].

The expression of *DNAJC19*, *DNM1L*, *LRPPRC*, *SLC25A12*, *SLC25A14*, *SLC25A24* and *TOMM20* were consistently reduced in at least two of the brain regions of autism patients compared to controls (Table 2).

None of the *P* values of altered expression of genes withstand multimarker testing (conventional Bonferroni approach).

### Genetic association study

Power analysis showed that the AGRE sample size of 841 families provided 37.3% and 95.5% power to detect an odds ratio of 1.2 and 1.5 respectively, for an allele frequency of 0.156 at an α of 0.05. However, the sample size of Japanese trios (188 trios) was underpowered to detect an association.

**Figure 2:**
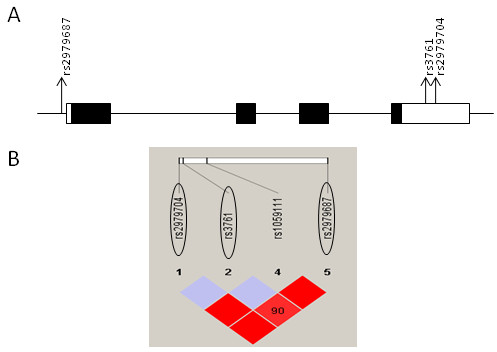
**Linkage disequilibrium (LD) plot of *NEFL.*** (**A**) Genomic structure of *NEFL* showing the locations of SNPs (denoted by arrows) used in the association study of AGRE samples. Exons are indicated by boxes, with translated regions in closed boxes and untranslated regions in open boxes. (**B**) LD structure of *NEFL* in Caucasian samples, based on D^′^ values. Tag single nucleotide polymorphisms (SNPs) are encircled.

*NEFL* showed a nominal association with autism in the AGRE samples (Table 3; Figure 2). The SNP rs2979704 in the untranslated region (UTR) of exon 4 showed a significant association with autism (*P* = 0.038; Z-score 2.066). After multimarker testing, there was a tendency for association (*P* = 0.083). No significant haplotype association was observed. There was no association of *NEFL* with autism in the Japanese samples (see Additional file 5.1).

**Table 3:** **FBAT analysis of*NEFL*in AGRE family samples**

**Marker**	**Location**	**Allele**^**a**^	**Families**^**b**^	**Frequency**	**Z-score**^**c**^	***P*value**^**d**^
rs2979704	Exon 4 UTR	T	356	0.844	2.066	***0.038***
		C		0.156	−2.066	
rs3761	Exon 4 UTR	G	291	0.894	−1.655	0.097
		A		0.106	1.655	
rs2979687	5’	C	583	0.654	0.859	0.390
		T		0.346	−0.859	
*P* value after multimarker testing						0.083

^a^Major allele is listed first; ^b^Number of informative families used by FBAT; ^c^Positive score indicates the risk allele, negative score indicates the protective allele; ^d^*P* <0.05, additive model, significant *P* values are indicated in bold italic; UTR, Untranslated region.

*SLC25A27* showed a nominal association with autism in Japanese samples (Table 4). The SNP rs6901178 in intron 4 showed an association with autism (*P* = 0.046; Z-score 1.990. After multimarker testing, there was a tendency for association (*P* = 0.073). No significant haplotype association was observed. There was no association of *SLC25A27* with autism in the AGRE samples (see Additional file 5.2).

**Table 4:** **FBAT analysis of*SLC25A27*in Japanese family samples**

**Marker**	**Location**	**Allele**^**a**^	**Families**^**b**^	**Frequency**	**Z-score**^**c**^	***P*value**^**d**^
rs12192544	5’	C	68	0.888	0.000	1.000
		G		0.112	0.000	
rs9381469	Intron 3	G	138	0.532	0.147	0.883
		A		0.468	−0.147	
rs6901132	Intron 4	A	132	0.573	0.899	0.368
		G		0.427	−0.899	
rs6901178	Intron 4	G	61	0.896	−1.990	***0.046***
		A		0.104	1.990	
rs2270450	Exon 9 UTR	C	109	0.771	0.877	0.380
		T		0.229	−0.877	
*P* value after multimarker testing						0.073

^a^Major allele is listed first; ^b^Number of informative families used by FBAT; ^c^Positive score indicates the risk allele, negative score indicates the protective allele; ^d^*P* <0.05, additive model, significant *P* values are indicated in bold italics; UTR, Untranslated region.

*MTX2* did not show any significant association with autism in the AGRE or Japanese samples (see Additional files 5.3 and 5.4).

## Discussion

Our study of MtD in autism involves a wide array of genes related to diverse mitochondrial functions. We report brain region-specific alterations in the expression of these genes in autism. *MTX2*, *NEFL* and *SLC25A27* showed consistently reduced expression in the ACG, MC and THL of autism patients. We also observed nominal genetic association of *NEFL* and *SLC25A27* with autism.

Gene expression was analyzed in three brain regions: ACG, MC and THL. ACG has been found to be involved in emotion formation and processing, learning and memory [27,28]; MC in planning, control and execution of voluntary motor functions [29]; and THL in the processing and relaying of sensory information [30]. In autistic individuals, abnormalities of anterior cingulate have been found to be linked with impairments in cognitive control [31], social orientation [32], social target detection [33], and response monitoring [34]. Increased white matter volume of MC has been reported to be associated with motor impairments in autistic children [35]. Impairments in auditory, tactile, and visual sensory stimuli processing, found in autistic individuals, have been attributed to THL abnormalities [36]. Reduced thalamic volume has also been observed in autism [37].

Brain samples from the cerebellum and cortices had been used in previous studies of MtD in autism. The brain regions (ACG, MC and THL) used in our study have not been reported elsewhere. The differences in the results of our study and other whole genome transcriptomic analyses of autism brain [17,38,39] might be due to the differences in the regions of brain that were analyzed. The differences in metabolic demands or brain region-specific pathophysiology could affect the expression of mitochondrial genes. The etiological heterogeneity and criteria for sample selection might also have influenced the results since MtD is observed in only a subset of autistic individuals.

*NEFL*, *SLC25A27* and *MTX2* showed reduced expression in all the three brain regions of autism patients. *NEFL* is located in 8p21.2, which has been suggested as a susceptible region for autism in a genome-wide association study [40]. Moreover, 8p is known as a potential hub for developmental neuropsychiatric disorders [41]. Being a major constituent of neurofilaments, NEFL plays a pivotal function in the assembly and maintenance of axonal cytoskeleton [42]. Knocking out of *Nefl* has been found to reduce axonal caliber and conduction velocity in mice [43]. Sensorimotor impairments and reversal learning deficits have been observed in *Nefl* transgenic mice [44]. NEFL has also been found to have a vital role in regulating mitochondrial morphology, fusion, and motility in neurons [45,46]. Reduced NEFL expression may thus restrict mitochondrial translocation to areas of the cell requiring energy. We observed a nominal association of an *NEFL* SNP with autism in the AGRE samples. However, this SNP is located in the UTR of exon 4 and might not have a functional significance.

SLC25A27, also known as uncoupling protein 4 (UCP4), belongs to the large family of mitochondrial anion carrier proteins that are located on the inner mitochondrial membrane. It is expressed predominantly in the central nervous system (CNS) [47]. It has also been suggested to have roles in the reduction of reactive oxygen species [48], neuroprotection against OS and ATP deficiency [49], inhibition of apoptosis [50], neuronal cell differentiation [51], mitochondrial biogenesis [52], and mitochondrial calcium homeostasis [53]. Downregulation of *SLC25A27* could thus have detrimental effects on these processes. Pharmacological targeting of neuronal uncoupling proteins (UCPs) represents an important avenue to combat MtD. Fatty acids have been reported to activate UCPs [54,55]. Consequently, a ketogenic diet has been found to increase the protein levels and activities of UCPs, including that of SLC25A27 [56]. We observed a nominal association of *SLC25A27* with autism in Japanese samples. However, rs6901178, the SNP that showed association, is located in intron 4 and might not have a functional significance.

MTX2, located on the cytosolic face of the outer mitochondrial membrane, has been suggested to function as an import receptor for mitochondrial preproteins, a crucial process for cell survival [57,58]. It also plays a major role in the regulation of apoptosis [59]. In this study, we observed a downregulation of *MTX2* in the ACG, MC and THL of autism patients; however, we did not observe an association of this gene with autism.

We also observed, in autism brains, region-specific alterations in the expression of several other mitochondria-related genes (Table 2). These genes fall into the ten functional groups as described in Additional file 3 and presented below:

1) *Membrane polarization and potential (MPP)*: MPP plays a crucial role in energy production, maintenance of calcium homeostasis, protein import and cell survival [60,61]. We observed, in the ACG of autism patients, an elevated expression of *BCL2* and *TP53*, which are involved in the maintenance of MPP.

2) *Mitochondrial transport*: In brain, the proper localization of mitochondria in the neurons is necessary for the generation of synaptic and action potentials, regulation of intracellular calcium dynamics and ATP synthesis [62,63]. In various regions of autism brains, we observed alterations in the expression of several genes related to mitochondrial transport, such as, *AIP*, *BCL2*, *DNAJC19*, *HSP90AA1*, *MFN2*, *MIPEP*, *TP53* and *TSPO*. The expression of *DNAJC19* was downregulated in the MC and THL of autism patients.

3) *Small molecule transport, SLC25A family*: The expression of several members of SLC25A solute carrier family was altered, with most of them being downregulated, in autism. Mitochondrial solute carriers transport a variety of solutes (di- and tri-carboxylates, keto acids, amino acids, nucleotides and coenzymes/cofactors) across the inner mitochondrial membrane [64]. We observed a reduced expression of *SLC25A12* and *SLC25A14* in the ACG and MC of autism patients. However, upregulated expression of *SLC25A12* has been observed in some prior studies [18,19]. The brain regions used in this study were different from those in the aforementioned studies. The variation in metabolic demands of different brain regions could consequently affect the expression of mitochondrial genes. There are also conflicting reports about the association of *SLC25A12* with autism [65-67]. The expression of *SLC25A24* was reduced in the MC and THL of autism patients.

4) *Targeting proteins to mitochondria.*

5) *Mitochondria protein import*: Of the hundreds of proteins that are found within the mitochondria, the mitochondrial genome encodes only 13, and the rest must be imported from the cytosol [68]. The nuclear-encoded, cytoplasmically synthesized proteins should be precisely targeted and imported to the mitochondria. In this study, the expression of several genes involved in protein targeting and import were found to be altered, with the majority of them being downregulated, in autism brains. Among these, *DNAJC19* was downregulated in the MC and THL of autism patients.

6) *Outer membrane translocation.*

7) *Inner membrane translocation*: The TIMM/TOMM translocases are involved in the translocation of nuclear DNA-encoded mitochondrial proteins across the outer and inner mitochondrial membranes [69]. Several genes belonging to the TIMM/TOMM family showed altered expression in autism brain. *TOMM20* showed a reduced expression in the ACG and MC of autism patients.

8) *Mitochondrial fission and fusion*: The expression of *MFN2*, one of the genes involved in the regulation of mitochondrial fission and fusion was found to be downregulated in the ACG of autism patients. Mitochondrial fission and fusion are crucial in maintaining the integrity of mitochondria, electrical and biochemical connectivity, turnover of mitochondria, segregation and protection of mtDNA, and programmed cell death [70]. In the neurons, this is involved in the formation and function of synapses in the dendritic spines and axons [71,72].

9) *Mitochondrial localization*: We observed reduced expression of *DNM1L*, *LRPPRC*, *MFN2* and *RHOT2*, localized predominantly in the mitochondria. These genes are involved in the biogenesis, maintenance of morphology and integrity, trafficking, and homeostasis of mitochondria [73-75]. The expression of *DNM1L* and *LRPPRC* were reduced in the ACG and MC of autism patients.

10) *Apoptosis*: The expression of apoptotic genes were altered, with most of them being upregulated, in the brain of autism patients. Recent studies have demonstrated a possible association between neural cell death and autism [76,77].

A two-way ANOVA showed that the expression of all the genes that were differentially expressed in two or more brain regions of autism were dependent on the disease status rather than being region-specific (data not shown).

It is not yet clear if MtD is the cause or effect of autism. ASD patients have often been found to manifest biochemical or neuropathological traits linked with altered mitochondrial function. Since mitochondrial abnormalities often result in CNS dysfunction, leading to developmental regression, learning disability, and various behavioral disturbances, ASD could be an important clinical presentation of MtD [78]. However, the clinical features, and the biochemical and genetic abnormalities in ASD patients with an underlying MtD have been found to be heterogeneous. In addition, several of the biochemical abnormalities indicative of MtD may occur in the absence of any relevant genetic alterations [79]. On the contrary, mitochondrial abnormalities might also manifest as a secondary to certain pathophysiological processes involved in autism, such as immune dysregulation, OS and altered calcium homeostasis [79]. Even though it is possible that a greater proportion of individuals with ASD might have MtD at the genetic level, it may not be manifested clinically.

We observed only nominal association of *NEFL* and *SLC25A27* with autism. Recent studies have indicated that only a subset of autism may be associated with the biochemical endophenotype of mitochondrial energy deficiency [80]. Therefore, related genes might not show a strong association with the disorder. Considering the highly heterogeneous nature of autism, nominal associations of genes with subtle effects on the disease phenotype should not be ignored. The small sample size of the Japanese trios is, however, a serious limitation of this study. *MTX2*, *NEFL* and *SLC25A27* were selected for genetic association studies since their expression was reduced in all of the three brain regions analyzed. Nevertheless, there would have been other important genes directly impacting mitochondrial functions, albeit differential expression in just one or two brain regions of autism patients. However, a detailed study involving several genes was not possible due to financial constraints.

Factors inherent in postmortem brain studies, and beyond the investigator’s control, might have influenced our results. We did not have sufficient data regarding brain pH. However, large-scale gene analysis have shown that brain pH or PMI has no significant correlation with RNA integrity [81,82]. The pH could be lower in the postmortem brains of individuals who suffered prolonged agonal states, such as in respiratory arrest, multi-organ failure and coma [83]. However, the cause of death was sudden for most of the subjects included in our study. So, we assume that brain pH might not have affected the gene expression. The other concern is the effect of medication; antidepressants, antipsychotics and selective serotonin re-uptake inhibitors are known to inhibit mitochondrial activities [84,85]. In this study, medication status was available for only three autism patients, two of whom had received more than two classes of drugs (drug doses unknown). Therefore, it was difficult to examine the effects of medication on gene expression. Another matter of concern is that the cause of death for a majority of the autism patients was seizure or drowning, where the latter could also have been due to seizures. Seizure activity has been known to impair mitochondrial energy production by altering the activity of mitochondrial enzymes involved in ATP production [86,87]. In this study, we have not examined the expression of any genes directly involved in mitochondrial energy production. Therefore, we assume that the cause of death might not have influenced our results. Moreover, it is not yet clear if MtD is the cause or effect of seizures.

## Conclusions

Our study, though preliminary, brings to light some new genes associated with MtD in autism. Dysfunction of these genes could lead to defects in mitochondrial activities, including energy metabolism, thus augmenting and disseminating several brain abnormalities related to autism.

## Abbreviations

ACG: anterior cingulate gyrus; ADI-R: autism diagnostic interview-revised; AGRE: Autism Genetic Resource Exchange; ASD: autism spectrum disorders; CNS: central nervous system; Ct: threshold cycle; GDC: genomic DNA control; LD: Linkage disequilibrium; MC: motor cortex; MPP: membrane polarization and potential; MtD: mitochondrial dysfunction; OS: oxidative stress; PDD-NOS: pervasive developmental disorder-not otherwise specified; PMI: postmortem interval; PPCs: positive PCR controls; qPCR: real-time PCR; RTCs: reverse transcription controls; SCID: Structured Clinical Interview for DSM-IV; SNP: single nucleotide polymorphism; THL: thalamus; UCPs: uncoupling proteins; UCSC: University of California Santa Cruz; UTR: untranslated region.

## Competing interests

The authors declare that they have no competing interests.

## Authors’ contributions

AA was involved in conception, design, conducting experiments, data analysis and drafting of article. IT, KY, YI and TT were involved in analysis and interpretation of data. HM, TM, SY, MT, KJT, KM, YIwata, KS and HI were involved in drafting the article. KN, TS, TY and NM were involved in revising the article critically. All authors read and approved the final manuscript.

## Supplementary Material

Additional file 1 Demographic characteristics of postmortem brain samples. Click here for file

Additional file 2 **Human Mitochondria RT**^**2**^**Profiler**^**TM**^**PCR Array: Gene Table.** Description of genes included in the array. Click here for file

Additional file 3 **Human Mitochondria RT**^**2**^**Profiler**^**TM**^**PCR Array: Functional Gene Groupings.** Classification of genes according to their functions. Click here for file

Additional file 4 SNPs selected for the genetic association study. Click here for file

Additional file 5: **FBAT analysis.** Results of FBAT analysis of genes without significant association. Click here for file
