# Supplementary material for: Brain region-specific altered expression and association of mitochondria-related genes in autism
Source: Mol Autism. 2012 Nov 1;3:12. doi: 10.1186/2040-2392-3-12 (PMC3528421; doi:10.1186/2040-2392-3-12)
Supplement: Additional file 1 — Demographic characteristics of postmortem brain samples. [file 2040-2392-3-12-S1.pdf]

Demographic characteristics of postmortem brain samples

Brain region	Age (years; mean ± SD)			Postmortem interval (hours; mean ± SD)			Gender		
	Control	Autism	p-value[1]	Control	Autism	p-value[1]	Control	Autism	p-value[2]
ACG	13.40 ± 4.45	16.38 ± 8.33	0.345	15.60 ± 6.20	18.02 ± 14.52	0.643	6M, 4F	6M, 2F	0.638
MC	15.63 ± 6.74	17.43 ± 8.40	0.652	18.13 ± 2.90	18.86 ± 15.71	0.899	5M, 3F	5M, 2F	0.573
THL	15.56 ± 6.31	16.38 ± 8.33	0.821	17.44 ± 3.40	18.02 ± 14.52	0.909	6M, 3F	6M, 2F	0.563

[1]t-test
[2]Fisher's exact test
ACG: Anterior cingulate gyrus; MC: Motor cortex; THL: Thalamus
M: Male; F: Female
