# Supplementary material for: Brain region-specific altered expression and association of mitochondria-related genes in autism
Source: Mol Autism. 2012 Nov 1;3:12. doi: 10.1186/2040-2392-3-12 (PMC3528421; doi:10.1186/2040-2392-3-12)
Supplement: Additional file 4 — SNPs selected for the genetic association study. [file 2040-2392-3-12-S4.pdf]

**SNPs selected for the genetic association study**

| **Gene** | ***MTX2*** | | ***NEFL*** | | ***SLC25A27*** | |
|---|---|---|---|---|---|---|
| | **AGRE** | **Japanese** | **AGRE** | **Japanese** | **AGRE** | **Japanese** |
| **SNPs** | rs6720043<br>rs1403996<br>rs17806377<br>rs6748172<br>rs2103108 | rs6709357<br>rs17806377<br>rs6433582<br>rs1403993 | rs2979704<br>rs3761<br>rs2979687 | rs1059111 | rs12192544<br>rs3757241<br>rs953062<br>rs9296505<br>rs10498770 | rs12192544<br>rs9381469<br>rs6901132<br>rs6901178<br>rs2270450 |
