# Supplementary material for: Brain region-specific altered expression and association of mitochondria-related genes in autism
Source: Mol Autism. 2012 Nov 1;3:12. doi: 10.1186/2040-2392-3-12 (PMC3528421; doi:10.1186/2040-2392-3-12)
Supplement: Additional file 5: — FBAT analysis. Results of FBAT analysis of genes without significant association. [file 2040-2392-3-12-S5.pdf]

5.1: FBAT analysis of *NEFL* in Japanese family samples

Marker	Location	Allele[1]	Families[2]	Frequency	Z-score[3]	p-value
rs1059111	Exon 4 UTR	A	128	0.632	1.470	0.141
		T		0.368	-1.470	

[1] Major allele is listed first
[2] No. of informative families used by FBAT
[3] Positive score indicates the risk allele; negative score indicates the protective allele
UTR: Untranslated region

5.2: FBAT analysis of *SLC25A27* in AGRE family samples

**Marker**	**Location**	**Allele**[1]	**Families**[2]	**Frequency**	**Z-score**[3]	**p-value**
rs12192544	5’	C	472	0.776	0.434	0.664
		G		0.224	-0.434	
rs3757241	Exon 2	C	410	0.821	1.179	0.238
		T		0.179	-1.179	
rs953062	Intron 2	A	592	0.642	-0.769	0.442
		G		0.358	0.769	
rs9296505	Intron 3	A	628	0.530	0.186	0.853
		G		0.470	-0.186	
rs10498770	Intron 8	C	235	0.901	1.509	0.131
		G		0.099	-1.509	

[1] Major allele is listed first
[2] No. of informative families used by FBAT
[3] Positive score indicates the risk allele; negative score indicates the protective allele

5.3: FBAT analysis of *MTX2* in AGRE family samples

Marker	Location	Allele[1]	Families[2]	Frequency	Z-score[3]	p-value
rs6720043	Intron 2	A	478	0.752	0.741	0.458
		G	478	0.248	-0.741	
rs1403996	Intron 2	A	497	0.722	-0.866	0.386
		G	497	0.278	0.866	
rs17806377	Intron 2	T	547	0.728	0.784	0.433
		A	547	0.272	-0.784	
rs6748172	Intron 4	G	404	0.826	1.113	0.265
		A	404	0.174	-1.113	
rs2103108	Intron 10	C	600	0.584	-0.508	0.612
		G	600	0.416	0.508	

[1] Major allele is listed first
[2] No. of informative families used by FBAT
[3] Positive score indicates the risk allele; negative score indicates the protective allele

5.4: FBAT analysis of *MTX2* in Japanese family samples

Marker	Location	Allele[1]	Families[2]	Frequency	Z-score[3]	p-value
rs6709357	Intron 2	T	110	0.779	-1.209	0.226
		C		0.221	1.209	
rs17806377	Intron 2	T	89	0.849	1.675	0.094
		A		0.151	-1.675	
rs6433582	Intron 4	T	111	0.778	-1.119	0.263
		G		0.222	1.119	
rs1403993	Intron 10	G	126	0.538	-0.077	0.939
		A		0.462	0.077	

[1] Major allele is listed first
[2] No. of informative families used by FBAT
[3] Positive score indicates the risk allele; negative score indicates the protective allele
